# Professors John Bell, and Samuel Jackson

**Published:** 1851-10

**Authors:** 


*Professors John Bell, and Samuel Jackson.*—We alluded, many months since, to a paper controversy, conducted with much asperity, by the distinguished gentlemen whose names are prefixed. We have omitted heretofore to state, that a printed document was received, some time since, containing mutual explanations, and an amicable adjustment of all the points at issue between them. They have redeemed the example of pugnacity, which was very generally deprecated by the medical press, in thus affording an illustration of the virtue of mutual forgiveness. Quarrels, to say the least of them, are but sorry episodes in a life, the comforts and enjoyments of which may in no small measure be enhanced by mutual forbearance, kindness, and good will; and of all men, the votaries of the *healing* art should be expected to be lovers of peace.

